# Implementation of a Healthcare of Elderly Course With Multi-Professional Teachers for Undergraduate Medical Students in a Public University in Malaysia—A Quasi-Experimental Pre and Post Study

**DOI:** 10.3389/fpubh.2021.743804

**Published:** 2021-11-11

**Authors:** Zhi Ling Ng, Hazwan Mat Din, Nor Fadhlina Zakaria, Liyana Najwa Inche Mat, Wan Zul Haikal Wan Zukiman, Anim Md Shah, Ummi Nadira Daut, Hakimah Mohammad Sallehuddin

**Affiliations:** ^1^Department of Medicine, Faculty of Medicine and Health Sciences, Putra Malaysia University, Seri Kembangan, Malaysia; ^2^Malaysian Research Institute on Ageing, Putra Malaysia University, Seri Kembangan, Malaysia; ^3^Department of Neurology, Faculty of Medicine and Health Sciences, Putra University Malaysia, Seri Kembangan, Malaysia

**Keywords:** geriatric medicine, undergraduate, curriculum, Malaysia, competency

## Abstract

Clinical practise in the ageing population is changing from organ-specific specialty care to holistic care. This is done through comprehensive geriatric assessment and multi-disciplinary team management. Hence, we adopted an approach consisting of multi-professional teachers teaching a Healthcare of Elderly Course (HEC), in a public university in Malaysia. We aimed to analyze the students' attitude, self-perceived competency and interest in geriatric medicine as a career before and after the course. We also investigated variables that might influence this interest among these students. All 96 students in the course were invited to participate in the survey. Sixty-eight (70.8%) completed both pre and post-course questionnaires. Although most students (93%) have a positive attitude (University of California at Los Angeles Geriatric Attitude Scale >3) toward older adults at baseline, it did not significantly increase post-course. We found that the mean scores for self-perceived competency increased from 3.62 (±0.76) to 3.81 (±0.56) post-course (*p* < 0.01). However, the students remained neutral with no significant change in the mean interest in pursuing a geriatric medicine career after the course. Students with higher self-perceived competency post-course were found to be more interested in geriatric medicine (β = 0.56, *p* < 0.001). In conclusion, the HEC in our centre could sustain a positive attitude and increase self-perceived competency in students. It is important to increase the preparedness of our graduates in managing older adults with frailty and multimorbidity. Future studies may involve inter-professional education of students from multiple disciplines undergoing the same course to nurture real-life collaborations in managing the ageing population.

## Introduction

In a rapidly ageing Asia, where it is projected to be the home of more than half of the aged population of the world by 2050, developing a competent workforce in managing ageing and age-related diseases becomes an utmost priority (1). The first national survey on the teaching of geriatric medicine in Malaysian medical schools showed that the most prominent barrier to curriculum delivery was the lack of expertise, followed by the subject not being included in the curriculum. It was shown that none of the core geriatric competencies achieved a 100% coverage, and only a third of the participating medical schools had access to geriatricians (2). The gaps in teaching the subject further led to inadequate graduates being competent in geriatric medicine. It is a vicious cycle that needs to be intervened with meticulously planned approaches.

To develop more exposure for the learners and increase doctors with expertise in the field, the Programme Standards for Undergraduate Medical Education (3) has incorporated Geriatric Medicine as one of the core competencies. Furthermore, as developing countries have limited resources, an innovative and effective curriculum must be designed. Nevertheless, in developing a curriculum, consideration must be made on understanding the attitude and interests of medical students toward ageing and a career in geriatric medicine (4). The Malaysian Society of Geriatric Medicine (MSGM) has developed a recommended undergraduate curriculum for ageing and geriatric medicine tailored for Malaysians (5). The curriculum has been validated with understanding of the unique nature and needs of our ageing population. Validation was done through a rigorous Delphi exercise with experts who teach in academic institutions and clinical geriatricians who have worked with medical officers. This combination of experts helped to address the gaps between undergraduate education and real-life practise, and fulfil the principles of outcome-based education.

A dedicated course on ageing and geriatric medicine at the undergraduate level has been shown to improve the attitude and self-perceived competency of medical undergraduates in managing older adults with frailty and multimorbidity (6). To achieve this, the teaching approach on ageing and geriatric medicine often involves multi-professional teachers and applies innovations to ensure that students graduate with adequate competency (7).

The attitude of an individual toward older adults plays an important role in this specialty learning, partly because ageism has a considerable impact on medical practises toward older adults (8). Earlier this year, the United Nations had identified ageism as a global challenge and called for urgent actions for anti-ageism strategies. Ageism is simply defined as discrimination, stereotyping and prejudice against a person based on their age. This leads to financial loss through psychological, behavioural, and physiological change (9). It is ubiquitous with every one in two people being ageist, whereby this is primarily seen in the younger population, the male gender, and those of lower education background (10). Hence, there is a need to also assess this perception among our students undergoing the course.

The medical program at Universiti Putra Malaysia (UPM) is a 5-year program and leads to the conferment of the Doctor of Medicine (MD). Our institution did not have a geriatrician in the faculty for the last two decades, but this did not hinder students from an in-depth learning about this growing population. A Healthcare of Elderly Course (HEC) has been offered as a 3-week course for undergraduate medical students since 2000. Since its inception, this course was primarily taught by visiting geriatricians from the Malaysian Ministry of Health. The clinical bedside teaching was initially carried out in the Geriatric Unit, General Hospital Seremban, under the guidance of the current Head of National Geriatric Service, Dr. Yau Weng Keong. Until the recent COVID-19 pandemic, the students continued to have bedside teaching and learning on geriatric medicine in Hospital Kuala Lumpur. This hospital is the largest public hospital under the Ministry of Health to offer such service. The COVID-19 pandemic has dramatically changed the course's content and delivery, where it involved online lectures and inconsistent contact with patients whenever the Movement Control Order was enforced in the nation. This might cause tremendous stress and anxiety to our students (11), especially when they need to learn a multi-faceted course like ageing and geriatric medicine.

This paper discusses the change in attitude, self-perceived competency, and interest in a career in geriatric medicine among undergraduate medical students of Universiti Putra Malaysia before and after an HEC. The association between attitude and previous experience of taking care of older adults was investigated. Additionally, the influence of gender, ethnicity, experience in caring for older adults, attitude toward senior citizens, and self-perceived competency on the interest to pursue a career in geriatric medicine is also discussed.

## Materials and Methods

### Study Design

This was a Quasi-experimental without Control Groups, Pre, and Posttest study undertaken between August 2020 to November 2020 for a span of 14 weeks. All 96 medical students who underwent the 3-week HEC were invited to answer an online questionnaire before and within 2 weeks after completing the course. The curriculum allocated the students into four groups with 24 students in each group. Students rotated between Otorhinolaryngology (3 weeks), Ophthalmology (3 weeks), Family Medicine/Radiology (3 weeks), and Healthcare of Elderly (3 weeks) courses.

The 3-week HEC was embedded in the clinical year (third year) and comprised of three parts: lectures, practical sessions, and tutorials. The lectures were delivered by field experts over a week to cover various topics on ageing and geriatric medicine. These field experts were namely geriatricians, rehabilitation physicians, neurologists, occupational therapists, psycho-geriatricians, and lawyers. This was followed by a 2-week practical sessions of a geriatrician-led ward rounds, geriatric outpatient clinics, home visits, and occupational and physiotherapy clinics. The tutorial consisted of a series of seminars, where students presented and discussed healthy ageing and geriatric syndromes. At the end of the course, students were required to submit a logbook and sit for a theory examination.

Participants were aware that personal identifiable information was not collected within the questionnaire. Thus, participants remained anonymous and this encouraged truthfulness. As this course was conducted entirely in English and the students were required to attain a certain level of English proficiency before enrolling into the medical programme, their English command was adequate to complete the questionnaire.

### Measures

A written informed consent was provided by every participant in this study. Information on age, gender, ethnicity, experience in taking care of older adults in the family, and the interest to pursue geriatric medicine as a career were collected from all participants through the questionnaire. Attitude was assessed using a 14-item survey by the University of California at Los Angeles Geriatric Attitude Scale (UCLA-GAS) (12) and self-perceived competencies questionnaire from (6). These questionnaires were used due to their reliability.

The online questionnaires were developed using Google Forms, and the link was shared through WhatsApp and e-mail. Likert scales from 1 (strongly agree) to 5 (strongly disagree) was used to assess attitude and self-perceived competency, and from 1 (strongly interested) to 5 (strongly disinterested) to evaluate interest to pursue a career in geriatric medicine (Supplementary Material 1). Cronbach alpha for the UCLA-GAS and Self-Perceived Competency questionnaires were 0.668 and 0.975, respectively. This study was reviewed and approved by the Universiti Putra Malaysia Ethics Committee (JKEUPM-2020-320).

### Data Analysis

All measured variables were summarised to mean (standard deviation) for continuous variable and frequency (percentage) for categorical variable. For comparisons of means between before and after course completion, repeated measure of ANOVA was used. Analysis of within-subject effects was checked using repeated measure of ANOVA, namely the assumption of compound symmetry, normality of residuals, and homogeneity of variance. Linear regression was used to analyze the association between the following:

1) previous experience of taking care of an older adult and attitude, and
2) gender, ethnicity, experience in taking care of an older adult, attitude toward older adults and self-perceived competency, and the interest to pursue a career in geriatric medicine.

Attitude and competency scores were calculated according to the responses of the students. For positive statements, the scoring systems used for the responses were as follows: a score of 5 for strongly agree, a score of 4 for agree, a score of 3 for neutral, a score of 2 for disagree, and a score of 1 for strongly disagree. Reverse scoring was done for the negative statements. The maximum score for each positive attitude, perceived competency and interest, was 5. The *p* < 0.05 was considered as significant. Data analysis was done using IBM SPSS version 23.

## Results

### Sociodemographic Variables

Out of the 96 students, 68 completed both pre and post-course questionnaires, resulting in a response rate of 70.8%. Nearly 30% of students responded either to the pre or post-test questionnaire (incomplete response). This might be due to the changing situation during the pandemic, where a fraction of students could not attend clinical sessions during the Movement Control Order. Hence, the inability to complete the survey. The majority of students were female (65%, *n* = 45). The students were mostly of the Malay ethnic group (55.9%, *n* = 38), followed by Chinese (23.5%, *n* = 16), Indian (17.6%, *n* = 12), and other groups (2.9%, *n* = 2).

### Attitude Toward Older Adults

The mean pre-course UCLA attitude score was 3.41 (±.36), which indicated an overall positive attitude toward older adults, at commencement of the course. Only five students, or 7.4%, demonstrated scores (<3) consistent with a negative attitude toward older adults. The mean post-course UCLA attitude score reported a slight increment to 3.45 (±.35), which was not statistically significant (*p* = 0.299).

Table 1 shows means, standard deviations, and *p*-value of the attitudes of students toward older adults for each question in the UCLA-GAS. The statement most disagreed upon was “*The federal government should reallocate money to research on AIDS or paediatric diseases*.” The highly agreed statements (score >4) were for “*It is interesting listening to old people's accounts of their past experiences*” and “*It is society's responsibility to provide care for its older adults*.” Upon completion of the course, three students (4.4%) reported maintaining a negative attitude. None of the mean scores of individual components showed a significant change post-course.

**Table 1:** Students' means, standard deviations, and *P*-value of attitudes toward older people for each question.

**Attitude question**	**Mean(SD)**		**Mean difference (post-course–pre-course)^‡^**	***p*-value**
	**Pre-Course^†^**	**Post-Course^†^**		
1. Most old people are pleasant to be with	3.90 (0.81)	3.85 (0.78)	−0.04	0.625
2. The federal government should reallocate money to research on AIDS or paediatric diseases	2.04 (0.82)	2.10 (0.90)	0.06	0.583
3. If I have the choice, I would rather see younger patients than elderly ones	3.15 (0.82)	3.00 (0.91)	−0.15	0.206
4. It is society's responsibility to provide care for its elderly persons	4.41 (0.67)	4.38 (0.65)	−0.03	0.718
5. Medical care for old people uses up too much human and material resources	3.25 (0.90)	3.44 (1.04)	0.19	0.155
6. As people grow older, they become less organised and more confused	2.25 (0.82)	2.24 (0.87)	−0.02	0.888
7. Elderly patients tend to be more appreciative of the medical care I provide than are younger patients	3.66 (0.77)	3.69 (0.90)	0.03	0.771
8. Taking a medical history from elderly patients is frequently an ordeal	2.50 (0.78)	2.62 (0.99)	0.12	0.280
9. I tend to pay more attention and have more sympathy toward my elderly patients than my younger patients	3.74 (1.03)	3.72 (0.93)	−0.02	0.907
10. Old people in general do not contribute much to society	3.74 (0.91)	3.91 (0.82)	0.18	0.083
11. Treatment of chronically ill old patients is hopeless	3.85 (0.82)	4.00 (0.79)	0.15	0.214
12. Old persons don't contribute their fair share toward paying for their health care	3.53 (0.86)	3.68 (0.70)	0.15	0.221
13. In general, old people act too slow for modern society	3.35 (0.84)	3.29 (0.96)	−0.06	0.636
14. It is interesting listening to old people's accounts of their past experiences	4.43 (0.63)	4.44 (0.61)	0.02	0.843

† *Mean pre- and post-scores derived from the 5-point Likert scale evaluation instrument: 1 = strongly disagree; 5 = strongly agree*.

‡ *Difference = mean post-course scores—mean pre-course scores. SD, standard deviation*.

### Self-Perceived Competencies of Students

Self-perceived competency scores of students significantly improved upon completion of the course [mean score pre-course of 3.62 (±0.76) vs. post-course of 3.81 (±0.56); *p* = 0.009]. Fifty-nine students (86.8%) had a positive (>3) perception of their competency at the start of the course, and by the end of the course, sixty-six students (97.1%) had a positive perception of their competency. Table 2 shows the means, standard deviations, and *p*-value of the self-perceived rating scores of students for each competency question.

**Table 2:** Students' means, standard deviations, and *P*-value of self-perceived rating scores for each competency question.

**Competency question**	**Mean(SD)**		**Mean difference (post-course–pre-course)^‡^**	***P*-value**
	**Pre-Course^†^**	**Post-Course^†^**		
1. I feel competent to recognise, evaluate, and treat dementia in my older patients	3.50 (0.86)	3.66 (0.75)	0.16	0.078
2. I feel competent to recognise and minimise medication interactions for my older patients	3.51 (0.87)	3.68 (0.78)	0.16	0.132
3. I feel competent to recognise, evaluate, and treat acute delirium in my older patients	3.46 (0.89)	3.75 (0.70)	0.29	**0.001**
4. I feel competent to recognise, evaluate, and treat behavioural disturbances in my older patients with dementia	3.46 (0.92)	3.78 (0.69)	0.32	**0.001**
5. I feel competent to recognise, evaluate, and treat depression in my older patients	3.51 (0.92)	3.76 (0.67)	0.25	**0.016**
6. I feel competent to recognise, evaluate, and treat gait disturbances in my older patients	3.60 (0.92)	3.82 (0.71)	0.22	**0.035**
7. I feel competent to recognise, evaluate, and treat falls in my older patients	3.69 (0.85)	3.91 (0.64)	0.22	**0.018**
8. I feel competent to diagnose, evaluate, and treat various causes of urinary incontinence in my older patients	3.76 (0.90)	3.87 (0.71)	0.10	0.240
9. I feel competent to evaluate the decision-making capacity of my older patients	3.59 (0.89)	3.71 (0.65)	0.12	0.230
10. I feel competent to evaluate the cognitive function of my older patients	3.71 (0.83)	3.82 (0.65)	0.12	0.220
11. I feel competent to evaluate the functional capacity of my older patients	3.76 (0.83)	3.90 (0.60)	0.13	0.151
12. I feel competent to recognise when my older patient needs to transition to a more supportive living situation (such as assisted living or a skilled nursing facility)	3.76 (0.92)	3.96 (0.61)	0.19	0.074
13. I feel competent to choose/recommend and arrange my older patient's transition to a more supportive living facility (such as assisted living or a skilled nursing facility)	3.68 (0.91)	3.81 (0.68)	0.13	0.236
14. I feel competent to care for patients who reside in community care facilities	3.66 (0.78)	3.93 (0.63)	0.27	**0.008**

† *Mean pre- and post-scores derived from the 5-point Likert scale evaluation instrument: 1 = strongly disagree, 5 = strongly agree*.

‡ *Difference = mean post-course scores–mean pre-course scores. SD, standard deviation. P-value in bold indicates significant mean difference*.

The highest self-perceived post-course competency was noted for the statement “*I feel competent to recognise when my older patient needs to transition to a more supportive living situation (such as assisted living or a skilled nursing facility)*,” with a post-course mean score of 3.96. Meanwhile the statement with the lowest self-perceived post-course competency was “*I feel competent to recognise, evaluate, and treat dementia in my older patients*,” with a post-course mean score of 3.66. The statement “*I feel competent to recognise, evaluate, and treat behavioural disturbances in my older patients with dementia*” and “*I feel competent to recognise, evaluate, and treat acute delirium in my older patients*” showed the most significant improvement post-course, with a change in mean score of 0.32 and 0.29, respectively (*p* < 0.001).

### Interest in Geriatric Medicine as a Career Choice

Most students remained neutral toward having geriatric medicine as a career of choice, both in the pre-course (52.9%) and the post-course (61.8%). At commencement, 35.4% of students were either interested or strongly interested in having geriatric medicine as a career choice, while 11.8% were either disinterested or strongly disinterested. Post-course, 29.4% of the students showed interest, while 8.8% were disinterested, and none were strongly disinterested. However, there was no significant difference in the interest of geriatric medicine as a career of choice in the pre and post-course (*p* > 0.95).

### Experience in Taking Care of Older Adults

Among the 68 students, 48 (70.6%) had experience in taking care of older adults in their family. There was no significant association between attitude and experience in taking care of an older adult family member (*p* = 0.509).

### Association Between “Interest in Geriatric Medicine as a Career of Choice” and Study Variables

Among gender, ethnicity, experience in taking care of an older adult, attitude toward older adults, and self-perceived competency, we found that only self-perceived competency of students showed a good and significant association (β = 0.56, *p* < 0.001) with the interest of the student in a future career in geriatric medicine (Table 3).

**Table 3:** Association between Interest in geriatric medicine as a career of choice and study variables (post-course).

**Variable**	**β(SE)**	**95% CI**	***P*-value**
**Gender**			
Male (reference)	–	–	–
Female	0.14 (0.20)	−0.26, 0.53	0.497
**Ethnicity**			
Malay (reference)	–	–	–
Non-Malay	−0.06 (0.19)	−0.44,0.31	0.734
**Experience in caring for older people**			
No (reference)	-	-	-
Yes	0.20 (0.20)	−0.20,0.60	0.324
**Attitudes toward older people**	0.38 (0.26)	−0.14, 0.90	0.152
**Self-Perceived competency**	0.56 (0.17)	0.23, 0.90	**<0.001**

*P-value in bold indicates significant association*.

## Discussion

In this study, it was hoped to uplift the image of clinical practise toward the ageing population. Although attitude is one of the critical constructs of ageism, the specific course did not significantly improve attitude. The UCLA-GAS may screen for stereotyping and discrimination related to ageism with acceptable reliability on four dimensions of attitude: Social Value, Medical Care, Compassion, and Resource Distribution. Among the four dimensions, attitude toward medical care contributes the most to the overall attitude (13).

The attitude of a person is a complex, multidimensional, and challenging aspect to accurately quantify in medical education research. Nevertheless, to combat ageism among medical graduates, attitude toward older adults must be accurately quantified and understood, and this should be the future work in the field of geriatric education (14). A locally developed, validated, or modified instrument will tremendously help medical educators to effectively develop geriatric medicine curricula that foster positive attitude toward older adults and combat ageism as a core graduate outcome (5, 8). Even though our study did not show any association between previous experience of caring for an older family member and attitude toward an older adult, another study among senior medical students in a public university in Malaysia (*n* = 116) showed that having grandparents, was a significant association (*p* < 0.05) (15).

Our study highlighted that the self-perceived competency of students heightened after the course. This was in line with another study in Australia (6). Students perceived themselves to be most competent in managing transitions of care and least capable in recognising, evaluating, and treating dementia. The most remarkable improvement of self-perceived competencies was in managing behavioural symptoms of dementia, followed by identifying, evaluating, and treating acute delirium. A self-assessment among graduates tends to differ with seniority, with an over estimation among the younger age group. Therefore, this assessment should be taken alongside a formal assessment by the faculty to ensure congruence (16). Student self-perceived competencies can be considered as a credit to the current curriculum. They should be conducted on a regular basis to implement necessary interventions and to enhance professional competencies and the quality of care (17). It was previously found that many medical institutions and medical educators have used the self-assessment of students as one of the measures to fulfil an outcome-based education (18).

Increasing the workforce in geriatric care has been a focus in many research on geriatric education. Our study showed that a higher self-perceived competency score had a significant association with a higher interest in pursuing a career in geriatric medicine. This might be explained by the impact of a positive influence of the module toward a career choice in geriatrics (19). Our study also showed no association between previous experience and interest. This differed from a finding in another study which found that frequent contact with an older adult and undertaking courses in ageing significantly increased the interest of students in working with older adults (19, 20). Studies have shown that medical students did not have enough exposure to older adult patients during their education and, therefore, did not see the drawbacks of ageing with multimorbidity (21, 22). Hence, having a dedicated course, such as HEC, will increase their exposure, skills, and experience at the undergraduate level. It was also suggested that a more positive attitude had been linked to a willingness to pursue geriatric medicine as a career (23, 24). However, despite generally having a positive attitude toward older adults, no significant association was found concerning the interest of our students in geriatric medicine. Only 1 in 3 students in our study considered a future career in geriatric medicine, mirroring the findings in studies done in Singapore and Ireland (19, 25).

The training of the healthcare workforce should not only focus on the attainment of knowledge and skills in managing diseases pertinent in the ageing population, but also the ability to work in an interdisciplinary team (26, 27). Therefore, a more refined older adult-friendly curriculum is recommended to nurture enthusiasm in caring for older adults (28). Although the complexity in handling older adults was found to be a significant barrier to working in the field, the exposure to a course in ageing and geriatric medicine would help spark interests (29).

## Strength

This was the first study in Malaysia that assessed the attitude, self-perceived competencies, and interests of students in pursuing a geriatric medicine career before and after an HEC with multi-professional teachers at the undergraduate level. This study was carried out during the COVID-19 pandemic to assess if the change in the implemented teaching method can sustain a good attitude after the course and improve self-perceived competencies.

## Limitation

There a few limitations identified in this study. Firstly, the study was conducted during the COVID-19 pandemic, with limited bedside teaching in the ward and clinic. Only about half of the students could attend a clinic session with a geriatrician, while the other half received no clinical exposure. Secondly, this study had a small sample size and was conducted in a single public institution, thus limiting generalizability. Thirdly, only medical students in the early clinical years (i.e., third year) were involved in the study, thus, the findings may not apply to other different cohorts of health professionals. Finally, we did not have a control group in this study. Hence, the results may call for judicious interpretations.

## Conclusion and Recommendation

Our study has shown that implementing multi-professional teachers in an HEC could sustain a positive attitude and improve self-perceived competencies among the undergraduate medical students at our learning institute. However, increasing the enthusiasm in students for pursuing a geriatric medicine career alone was not sufficient as most students remained neutral after the course. Therefore, a more refined older adult-friendly curriculum that addresses the complexity of caring for older adults is recommended to encourage a future career in the field.

In an ageing society, the training of the healthcare workforce should not only focus on knowledge and skills in managing the diseases pertinent in the ageing group but also the ability to work in an interdisciplinary team. Although our course involves multi-professional teachers, it only partially exposes our students to the reality of managing an older adult as a team. We recommend that the course employ an inter-professional teaching method in the future, which may include nursing, pharmacy, law, dietetic, physiotherapy, speech therapy, occupational therapy, and social workers. Secondly, although the UCLA-GAS was internationally validated among medical students and health professionals, there is a need to consider the multicultural background of local students. Future studies should focus on a locally validated tool to measure the attitude toward older adults and perhaps construct against ageism related stereotyping and discrimination.

## Data Availability Statement

The raw data supporting the conclusions of this article will be made available by the authors, without undue reservation.

## Ethics Statement

The studies involving human participants were reviewed and approved by Universiti Putra Malaysia Ethics Committee JKEUPM-2020-320. The patients/participants provided their written informed consent to participate in this study.

## Author Contributions

HS, NZ, and UD: conceptualisation. HM and HS: methodology. HM and NZ: formal analysis. NZ, LI, and WW: investigation. HS: writing—original draught preparation of all sections. HS and AM: writing—original draught preparation of introduction. NZ and UD: writing—original draught preparation of introduction and discussion. HM and NZ: writing—original draught preparation of methods and results. HS, NZ, UD, AM, and WW: writing—final review and editing. All authors contributed to the article and approved the submitted version.

## Funding

The publication of this manuscript was supported by University Putra Malaysia Journal Publication Fund (VOT9001103).

## Author Disclaimer

The authors alone are responsible for the views expressed in this article. They do not necessarily represent the institutions' views, decisions, or policies in which they are affiliated to.

## Conflict of Interest

The authors declare that the research was conducted in the absence of any commercial or financial relationships that could be construed as a potential conflict of interest.

## Publisher's Note

All claims expressed in this article are solely those of the authors and do not necessarily represent those of their affiliated organizations, or those of the publisher, the editors and the reviewers. Any product that may be evaluated in this article, or claim that may be made by its manufacturer, is not guaranteed or endorsed by the publisher.
