# Supplementary material for: Implementation of a Healthcare of Elderly Course With Multi-Professional Teachers for Undergraduate Medical Students in a Public University in Malaysia—A Quasi-Experimental Pre and Post Study
Source: Front Public Health. 2021 Nov 11;9:743804. doi: 10.3389/fpubh.2021.743804 (PMC8632039; doi:10.3389/fpubh.2021.743804)
Supplement: Supplementary file 1 [file Data_Sheet_1.docx]

Supplement 1

Section A: Demographics and characteristics

1) Age: …………..

2) Gender : …………..

3) Ethnicity

i-Malay

ii-Other Bumiputera

iii-Indian

iv-Chinese

v- Others

4) Previous experience taking care of elderly (family/relatives)

Yes/No

Section B: Geriatric Attitude scale

1. Most old people are pleasant to be with.

2. The federal government should reallocate money to research on AIDS or pediatric diseases.

3. If I have the choice, I would rather see younger patients than elderly ones.

4. It is society’s responsibility to provide care for its elderly persons.

5. Medical care for old people uses up too much human and material resources.

6. As people grow older, they become less organized and more confused.

7. Elderly patients tend to be more appreciative of the medical care I provide than are younger patients.

8. Taking a medical history from elderly patients is frequently an ordeal.

9. I tend to pay more attention and have more sympathy towards my elderly patients than my

younger patients.

10. Old people in general do not contribute much to society.

11. Treatment of chronically ill old patients is hopeless.

12. Old persons do not contribute their fair share towards paying for their health care.

13. In general, old people act too slow for modern society.

14. It is interesting listening to old people accounts of their past experiences.

5-point Likert scale, 1= Strongly agree,2=Agree, 3=Neutral,4=Disagree, 5=Strongly disagree

Section C: Self-perceived Competence to Care for Older Adults Questionnaire

a) In providing medical care:

1. I feel competent to recognize, evaluate, and treat dementia in my older patients

2. I feel competent to recognize and minimize medication interactions for my older patients

3. I feel competent to recognize, evaluate, and treat acute delirium in my older patients

4. I feel competent to recognize, evaluate, and treat behavioral disturbances in my older patients with dementia

5. I feel competent to recognize, evaluate, and treat depression in my older patients

6. I feel competent to recognize, evaluate, and treat gait disturbances in my older patients

7. I feel competent to recognize, evaluate, and treat falls in my older patients

8. I feel competent to diagnose, evaluate, and treat various causes of urinary incontinence in my older patients

b) In assessing older patients:

1. I feel competent to evaluate the decision-making capacity of my older patients

2. I feel competent to evaluate the cognitive function of my older patients

3. I feel competent to evaluate the functional capacity of my older patients

c) In managing patients’ care transitions:

1. I feel competent to recognize when my older patient needs to transition to a more supportive

living situation (such as assisted living or a skilled nursing facility)

2. I feel competent to choose/recommend and arrange my older patient’s transition to a more

supportive living facility (such as assisted living or a skilled nursing facility)

3. I feel competent to care for patients who reside in community care facilities

5-point Likert scale, 1= Strongly agree,2=Agree, 3=Neutral,4=Disagree, 5=Strongly disagree

Section D: Interest in geriatric medicine as a career choice

5-point Likert scale, 1=strongly interested,2=interested,3=neutral,4=not interested, 5=strongly disinterested
